# Supplementary figures and images for: Stimulation of Distinct Rhizosphere Bacteria Drives Phosphorus and Nitrogen Mineralization in Oilseed Rape under Field Conditions
Source: mSystems. 2022 Jul 13;7(4):e00025-22. doi: 10.1128/msystems.00025-22 (PMC9426549; doi:10.1128/msystems.00025-22)

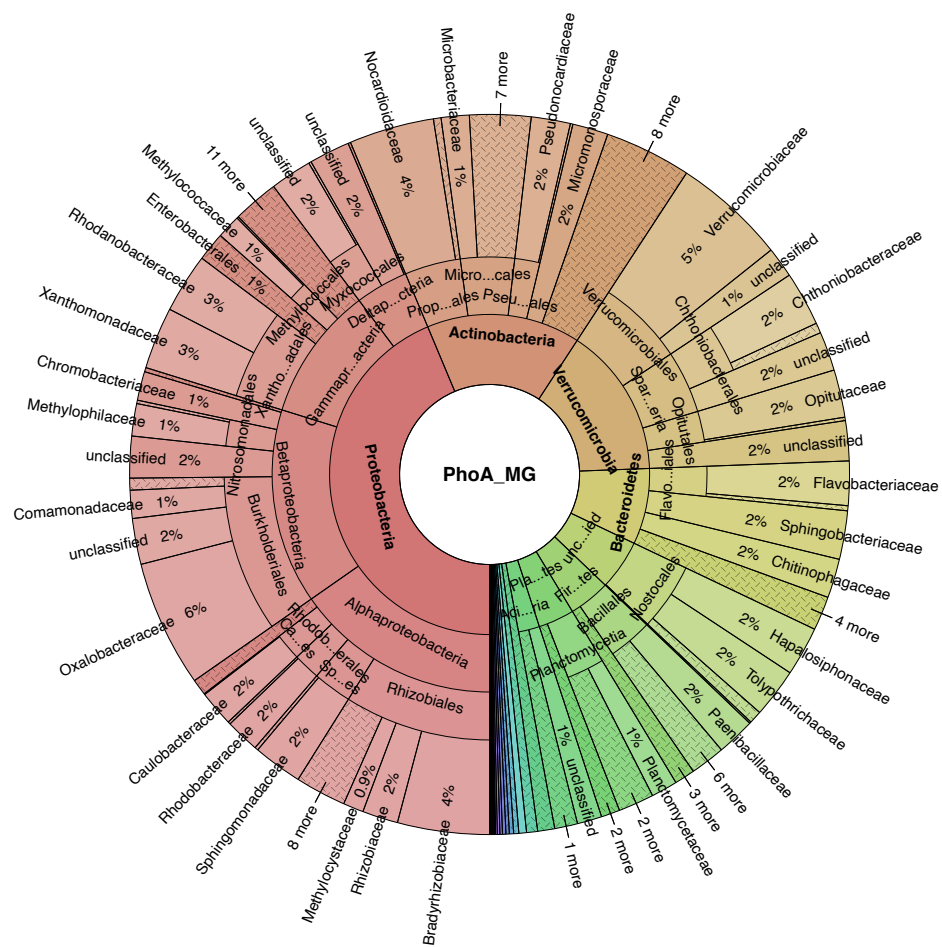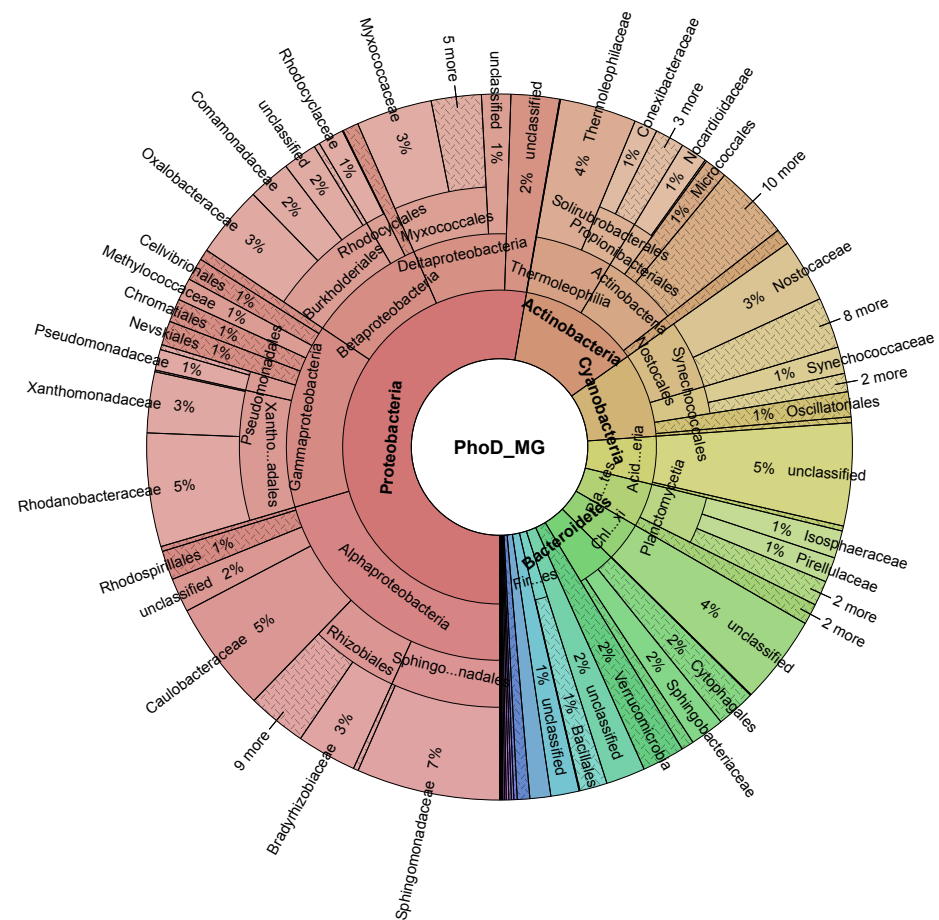

Supplement: FIG S7 [file msystems.00025-22-s0007.pdf]
